# Fish Nutritional Value as an Approach to Children's Nutrition

**DOI:** 10.3389/fnut.2021.780844

**Published:** 2021-12-15

**Authors:** Sahya Maulu, Kundananji Nawanzi, Mohsen Abdel-Tawwab, Hala Saber Khalil

**Affiliations:** ^1^Centre for Innovative Approach Zambia (CIAZ), Lusaka, Zambia; ^2^Department of Agriculture and Aquatic Sciences, Kapasa Makasa University, Chinsali, Zambia; ^3^Department of Fish Biology and Ecology, Central Laboratory for Aquaculture Research, Agriculture Research Center, Abbassa, Sharqia, Egypt; ^4^Aquaculture and Fisheries Group, Wageningen Institute of Animal Sciences, Wageningen University and Research, Wageningen, Netherlands; ^5^WorldFish, Africa Aquaculture Research and Training Center, Abbassa, Egypt; ^6^Aquaculture Division, National Institute of Oceanography and Fisheries (NIOF), Cairo, Egypt

**Keywords:** aquatic food, omega-3, nutrition, malnutrition, brain development, PUFAs, physiological functions

## Abstract

Fish is a relatively cheap and accessible source of animal protein for human consumption even in rural communities. It is critical for global food and nutrition security, and its consumption continues to increase. As a highly nutritious food, fish consumption is highly recommended for children and expectant mothers for normal growth and development. The present paper explores the nutritional value of fish as approach to nutrition in children and its benefits. The findings reveal that fish is a valuable source of essential amino acids (EAA) and polyunsaturated fatty acids (PUFAs) that play important physiological functions for maintenance and development of fetuses, neonates, and infant brains. Therefore, it could be a valuable tool in the fight against food insecurity and malnutrition. However, fish and fish products are also highly susceptible to contamination by various organic and inorganic compounds that threaten public health. Particularly, heavy metals and biogenic amines (BAs) have shown adverse effects when contaminated fish is consumed, and the effects in children have been worse. Hence, while fish consumption is highly recommended for children's nutrition, the safety and quality of the product should always be checked to safeguard public health.

## Introduction

Fish is consistently among the most commonly used and low-cost dietary sources of animal protein for most people worldwide (1, 2). It is a valuable source of essential nutrients, especially high-quality protein and fats (macronutrients), vitamins, and minerals (micronutrients) that make a vital contribution to the world's food and nutrition security (3). As a food product, fish is of greater importance in developing countries where it accounts for 75% of the daily animal protein, referred to as “rich and poor food” as an important companion (4, 5). Compared with other animal protein sources, fish is readily available even in poorer communities at a relatively cheaper price. Furthermore, fish production through aquaculture is considered sustainable and the most efficient way to produce high quality proteins for human consumption (6–8).

In children, inadequate intake of dietary protein could lead to serious health consequences, including stunted growth and poor development (9). Hence, the protein component of the human diet is very crucial and an important area of focus when it comes to malnutrition due to its physiological functions (9). Globally, malnutrition remains a major problem and it is estimated that 47 million children suffer from stunting, due to the lack of micronutrients of vitamin A, iron, and iodine, which is a source of public health concern in the world (10). Its consequences include nutritional blindness, impaired learning capabilities, poor growth, and increased morbidity and mortality rates (4). In many developing countries, malnutrition is a major risk for sickness and death in children (11). This is mainly driven by lack of access to high quality food products. Fisheries and aquaculture programs can address and mitigate issues of malnutrition in the world by increasing the access to fish (12, 13) due to its nutritional value. Therefore, increasing fish production could increase the access to fish products and improve the nutritional status in children which has the potential to end malnutrition and food insecurity.

Recently, the number of studies exploring the importance of fish consumption in children have increased. This is because fish has been recognized as an important source of high quality animal protein required for bodybuilding and other physiological functions in children compared to adults (14). Besides, during childhood stage, the provision of adequate protein intake is very crucial for the overall growth and development into adulthood. As fish is tender and easily digested than meat, its consumption in children would be an excellent source of calcium and fluorine essential for the development of strong bones and teeth (15). Furthermore, the consumption of fish, particularly oily fish, is essential for optimal development of the brain and neural system of the children, as omega-3 fatty acids in the form of docosahexaenoic (DHA) rather than alpha-linoleic acid (ALA) are required for optimal brain development (16). Unfortunately, much of the existing studies on fish have had a bias toward its economic importance and merely as a food item, while paying less attention to its nutritional value, particularly in children nutrition. Understanding the benefits associated with fish consumption is very cardinal for promoting the consumption of fish as it is often the cheapest source of animal protein in marginalized communities to improve the nutritional status normal development in children. This study, therefore, aimed to synthesize existing studies on the nutritional value of fish, including the benefits and risks associated with its consumption in children.

## Global Overview of Fish Consumption

**Figure 1:**
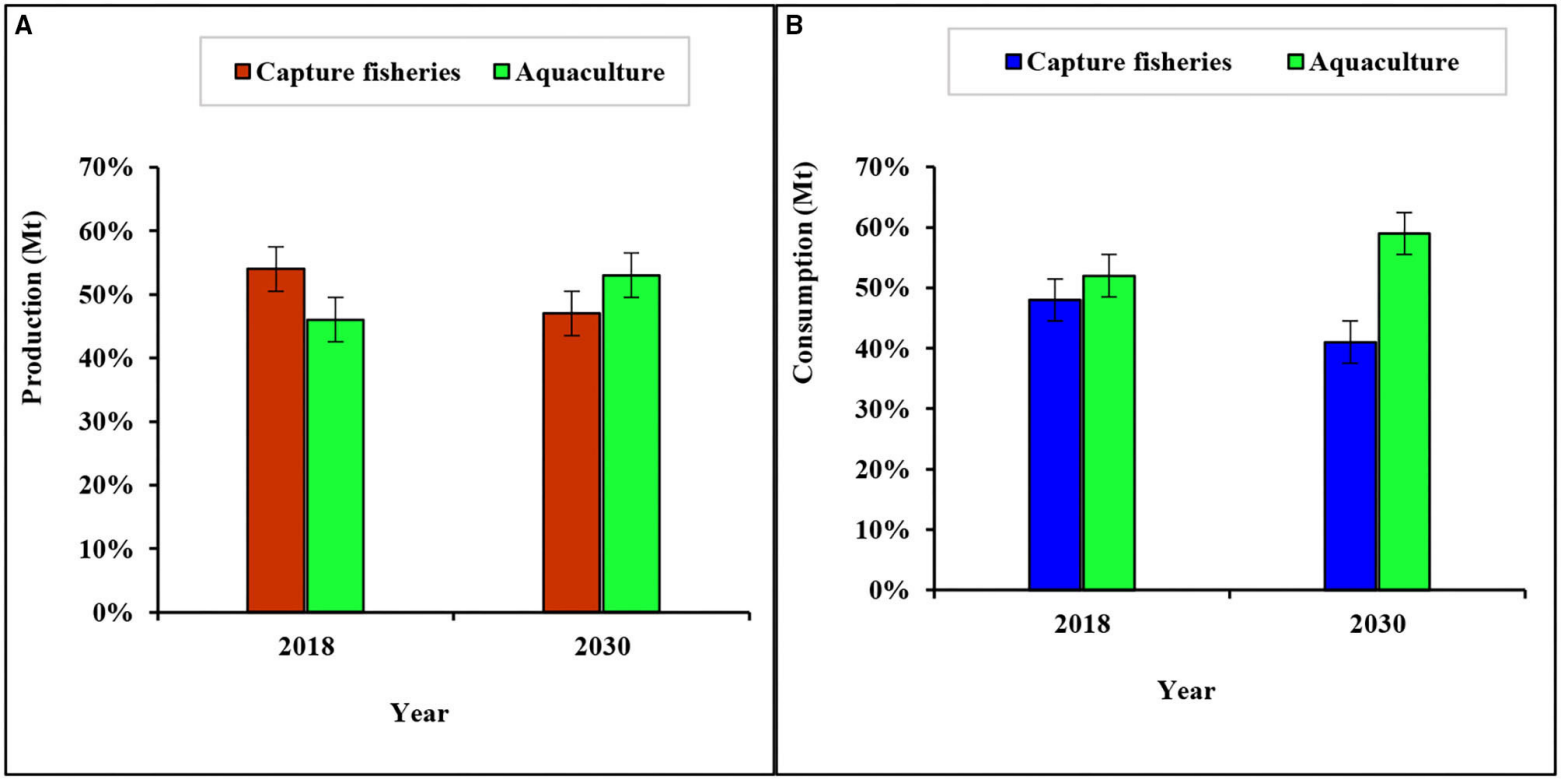
Global fish **(A)** production in 2018 and projected production in 2030, and **(B)** consumption in 2018 and projected consumption in 2030, from capture fisheries and aquaculture. Source: Adapted from FAO (3).

Fish is very crucial to a nutritious diet in many areas across the world and it provides about 3.3 billion people with almost 20% of their average per capita intake of animal protein. As the global population increases, potential nutritional concerns are raised, and fish represents an important source of animal protein. For this reason, global fish for human consumption is projected to increase by 16.3% indicating that 90% of the fish being produced will be utilized for human consumption by the year 2029 (17). In 2018, fish accounted for about 17% of the total animal protein and 7% of this was animal protein consumed globally (3). The consumption of fish and the fish products has experienced major changes in the past decades. The world evident per capita fish consumption has been increasing steadily from an average of 12.5 kg in the 80's to 14.4 kg in the 90's and reaching 20.5 kg in 2017 (18). This expansion in the consumption have been driven not only by the increase in production but also by the nutritional standards it has shown to provide to the people, reduced waste, better utilization, improved distribution channels, and increased demand (3). Therefore, the increase in the consumption globally is an indication that the health benefits of fish consumption are manifold and well-understood from both scientific and nutritional perspectives. This also means that fisheries and aquaculture will continue to play a very crucial role in meeting the animal protein demands of the global population, with aquaculture being the dominant supplier (Figure 1).

### The Chemical Composition of Fish

Fish contains 18–20% protein, and contains eight essential amino acids including sulfur containing lysine, methionine, and cysteine (12). It provides easily digestible protein of high biological value that is important for the growth and development of the body, the maintenance and repair of worn out tissues and for the production of enzymes and hormones necessary for many of the body's processes, it's contains less fat than red meat (19).

**Figure 2:**
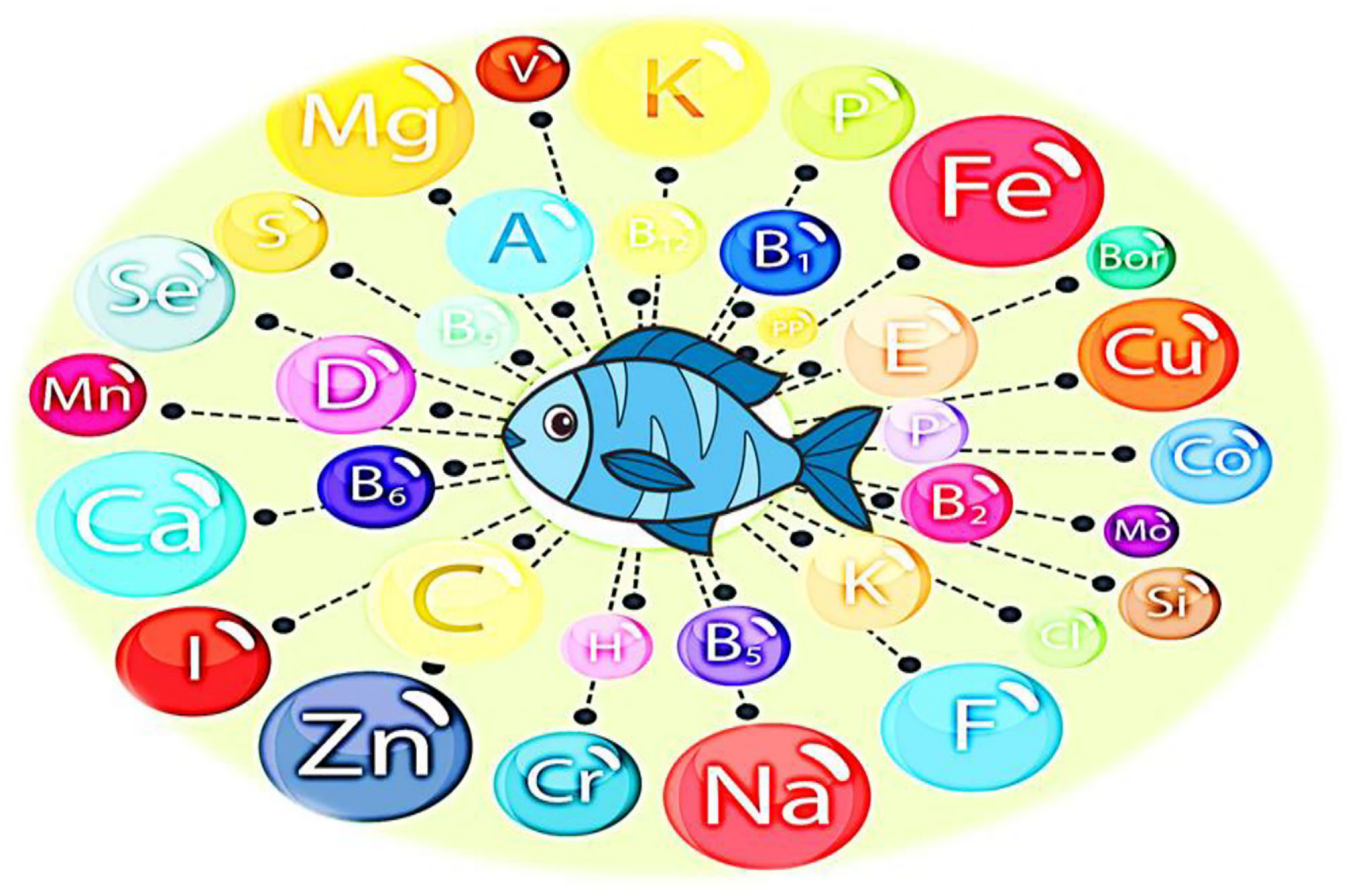
Fish as a vital source of vitamins and minerals for children.

The fat content ranges from 0.2 to 25%, especially polyunsaturated fatty acids (PUFAs), which are essential for the proper growth of children and are not associated with the occurrence of cardiovascular disease (20). Fats also contribute to the energy supply and aid in the adequate absorption of vitamins K, D, A, and E (21). Fish is a vital source of vitamins Figure 2, especially vitamins A and D of the fats, as well as thiamine, riboflavin, and niacin (vitamins B1, B2, and B3) (22). Vitamin A found in fish is more available in the body compared to plant foods and is essential for normal vision and bone growth, also, fatty fish contains more vitamin A than the lean types (22). Vitamin D, found in fish liver and oils, is essential for bone growth because, it is essential for calcium absorption and metabolism (23). Energy metabolism requires thiamin, niacin, and riboflavin (23). Fresh fish provides a small amount of vitamin C, which is essential for wound healing, maintaining the integrity of tissue, and assisting in the absorption of iron in the nervous system (22).

Phosphorus, calcium, iodine, iron, selenium, fluorine, and zinc are among the minerals found in fish and are extremely “bioavailable,” ensuring that they are readily consumed by the organism (24). Iron is critical for the formation of hemoglobin in the blood, which would be responsible for distributing oxygen across the body (24). Calcium is critical for the development and mineralization of bones, as well as the normal operation of tissues and the central nervous. It also plays a significant role in the clotting of blood (25). When young fish are consumed with their bones, the phosphorus, calcium, and fluorine consumption is greatest (25). Zinc is needed for growth performance, function of immune system and the maintenance of healthy skin (26). Iodine, found in aquatic food, is necessary for hormones that control body metabolism, growth and proper behavioral development in children (22). Fish clearly provides more to people's diets than just high-quality protein (27). As a result, fish can be a staple to every diet, avoiding starvation which, make these nutrients readily accessible to absorption by organs.

In recent decades, as people's concerns about their health have grown, so has their concern about fats (28). Fatty acids are molecules consisting of one glycerol and three fatty acids that serve as a source of energy in our body and are deposited in the meat, muscles and liver (29). When fat is ingested, lipase breaks it down into one glycerol and three fatty acids, while a few fatty oils molecules are pass through intact through the intestine (30). The ingested fat is at initial storage in the liver, the muscular or subcutaneous inner layer and then broken down as needed to provide energy (31).

Furthermore, saturated and unsaturated fatty acids are distinguished by the presence or absence of an intramolecular double bond (20, 32). Saturated fatty acids are found in animal oils and are harmful to an individual's health, while unsaturated fatty acids are found in vegetable oils and are beneficial to an individual's health (21, 33). As a result, polyunsaturated fatty acids (PUFAs), such as omega-3s, have received significant attention (12). There are several medications and able to pay foods on the market, and the number of products aimed at children has risen as well (34). However, since there is concern about indiscriminate and excessive PUFA intake, it is critical to understand the correct use of PUFAs (35). As a result, the purpose of this study is to investigate at the fundamental concepts, kinds of PUFAs, physiological mechanisms of action of PUFAs, and PUFA consumption in children.

### Polyunsaturated Fatty Acid Types

Animal oils, like pork, and butter, are rich in saturated fatty acids. Fish oil is an unsaturated fatty acid similar to animal oil (36). Unsaturated fatty acids make up the majority of vegetable fats, although saturated fatty acids like those found in coconut and palm oils are also present (37).

The persistent stability of saturated fatty acids causes them to harden and become white at cold temperatures. It is easy to store and does not easily strip when exposed to heat or pressure (38). Saturated fatty acids are problematic because they contribute to a number of circulatory and vascular issues (39). Saturated fatty acids, that harden at low temperatures, can cause atherosclerosis, angina, and stroke by raising cholesterol levels, and they can also alter blood flow (40). Some saturated fatty acid such as myristic acid C14/0, stearic acid C18/0, monounsaturated fatty acid as palmitoleic acid C16/1, Oleic acid C18/1 (41).

Unsaturated fatty acids on the other hand do not solidify and exist in liquid form at low temperatures due to their structural instability; they strip quickly when heat or pressure is applied, and spoil quickly (42). Unsaturated fatty acids are recognized to offer a variety of health benefits (43). Unsaturated fatty acids—linolenic acid (ALA) C18/3, linoleic acid C18/2, arachidonic acid (AA) C20/4; n−6, eicosapentaenoic acid (EPA) C20/5; n−3, and docosahexaenoic acid (DHA) C22/6; n−3 are those that have a physiologically vital role for children (44). The location of the first omega double bond, the carbon atom at the end of the carbon chain (the CH3 radical) within the fatty acid molecular structure, is used to classify unsaturated fatty acids (45). The essential fatty acids ALA, linoleic acid, and AA are required for optimal growth and health, but are not produced in animals' bodies, so they are classified as essential fatty acids (46). Wild fishes have higher levels of omega-3 PUFAs than farmed fish (47). Cold-water fishes contents contain greater amounts of long chain n3 PUFAs, which aid in their adaptation to the cold temperature (47). Aquatic oil, can provide EPA, Docosapentaenoic acid (DPA), DHA, and arachidonic acid (ARA), that can be used immediately in the body for regular physiological processes (20, 48). Omega-chain fatty acids are also known as unsaturated fatty acids (49).

Omega-3 (n-3), omega-6 (n-6), and omega-9 are all good examples (n-9) (50). Fish oil (for example, Sardine (10.14 EPA; 10.66 DHA), Menhaden (13.17 EPA; 8.56 DHA), Salmon (13.20 EPA; 18.23 DHA), Cod liver (9.90 EPA; 10.97 DHA), Herring (6.27 EPA; 4.21 DHA) and Fish such as Caviar, black and red (2.74 EPA; 3.80 DHA), values are g/100 g. (51). Vegetable oils (such as perilla oil, flaxseed oil, soybean oil, and canola oil) are both high in Omega-3. ALA, EPA and DHA are the nutritionally necessary omega-3 fatty acids (52, 53).

Animals have a limited ability to synthesize EPA and DHA (long-chain fatty acids) from ALA because they cannot produce omega-3 fatty acids (short-chain fatty acids) (54, 55). Grape seed oil, soybean oil, corn oil, sunflower oil, and cottonseed oil are high in omega-6 fatty acids (56, 57). Linoleic acid, linoleic acid, and AA are all omega-6 fatty acids (58). Oleic acids belong to the class of omega-9 fatty acids, and make up more than 80% of olive oil (59). Lard, palm oil, and sesame oil also contain omega-9 fatty acids (60).

#### PUFAs' Physiological Mode of Action

**Figure 3:**
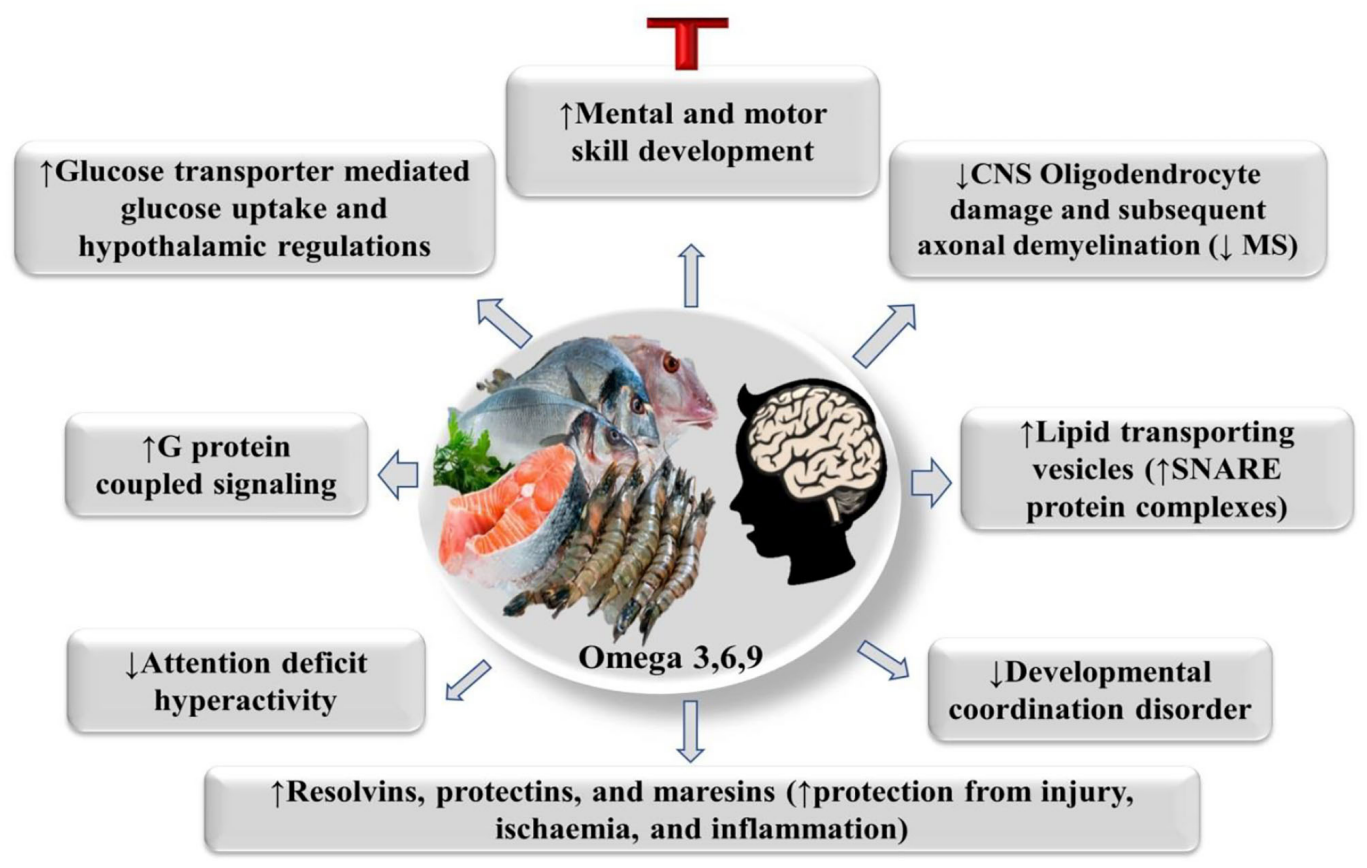
PUFAs' physiological mode of action on brain health. CNS, Central nervous system, G protein-coupled signaling (leading to altered gene expression).

PUFAs are necessary fatty acids that are not produced in animals' bodies but are required for optimal growth and health (61, 62). When PUFAs are deficient, a variety of symptoms can develop, therefore PUFAs are regarded medically essential (63). The positive health activities of DHA and EPA, which are the materials designated as omega-3 functionality, are particularly well-established (47, 55). EPA improves blood circulation and lowers cholesterol levels in the blood (low-density lipoprotein, LDL) (64, 65). DHA is a fatty acid present in brain tissue and the optic nerve, which assists to rejuvenate brain cells and improve brain function, Figure 3 (66–68). Omega-3 fatty acids, on the other hand, have anti-thrombotic, anti-arrhythmic, and anti-inflammatory properties, whereas omega-6 fatty acids are known to promote inflammation and thrombus development (69). DHA has been the most common n-3 LC-PUFA in the human nervous system (NS) (5 g in the human brain, 15% total fatty acids (FAs) (70). Additionally, DHA phospholipids have a great level of functional flexibility, which may be a key feature of a range of biochemical characteristics, including cognitive activities, acyl chain order, phase behavior, and synapse transmission locations of the synaptosomal cytoplasmic membrane in the brain has been preserved throughout growth and maintenance G protein-coupled signaling which leads to altered gene expression (71, 72). DHA also serves as an extra-and intracellular transcription factor, and blood DHA levels have been related to improved neurological development and visual in children (70, 73). EPA can stimulate the regeneration process of remyelination and cure multiple sclerosis of the central nervous system (CNS), after toxic damage to CNS oligodendrocytes (74). PUFAs' supplementation can help children with attention problems enhance their brain development and reading abilities (70, 75).

#### Consumption of PUFAs by Children

Omega-3 fatty acids are essential for children's health. Infants that were fed powdered formula with high grades of omega-3 fatty acid had better eye-hand coordination, attention, and social skills, as well as higher Intelligence Quotient (IQ) test results (76–78). It was also discovered that consuming long-chain polyunsaturated fatty acids (LCPUFAs) during pregnancy lengthened the pregnancy and lowered the frequency of premature deliveries (79, 80). According to Olsen et al. (81), children of moms who took fish oil during pregnancy had a lower risk of developing asthma in their teenage years. According to certain studies, powdered formula containing omega-3 helps preterm babies' growth and cognitive development (82, 83). The studies described above aren't conclusive; however they might be a cause to consume omega-3 that contains DHA or EPA. As a result, DHA and EPA are included in many powdered formulas now on the market. Furthermore, breast milk is the best supply of omega-3 (84), however it is heavily influenced by the diet that mothers consume (84). Nevertheless, according to a recent meta-analysis on omega-3 fatty acids, there is insufficient data to establish if LCPUFAs consumption during pregnancy aids cognitive or visual development (85).

### Food Fish Quality and Safety

The quality and safety of food products determines the protection of public health, social stability as well as the food and nutrition security (2, 86). Fish is vulnerable to contamination by pollutants such as heavy metals that threaten their safety for human consumption. Heavy metals are classified as elements having a high atomic weight and a density of at least five times greater compared to that of water and are present in nature from the earth's crust (87–90). Despite numerous heavy metal elements present in nature, mercury (Hg), arsenic (As), cadmium (Cd), chromium (Cr), and lead (Pb) are considered the most toxic elements and threat to public health. The widespread contamination of heavy metals in aquatic environment results mainly from anthropogenic activities including agricultural, industrial (such as mining), medical, and domestic applications (87, 90). Fish accumulate heavy metals by uptake through the gills and the skin when in contact and can bioaccumulate and bio-magnify them to toxic levels for human consumption (91, 92). However, the risks associated with consuming fish depends on the levels of contamination. The Food and Agriculture Organization (FAO) has set limits within which fish contaminated with heavy metals is considered safe for consumption (Table 1). As indicated, some elements could be toxic even at low levels while others at higher levels. Invariably, the consumption of fish and fish products contaminated with heavy metals at levels beyond safe limits could have adverse effects in humans. However, children are more vulnerable due to their low body weight and behavior. For example, exposure to Pb in children could cause learning deficit, intelligence quotient (IQ) lowering, and severe damages in the brain and kidneys (93). Consuming excess Cd levels in fish products could result in kidney failure and bones softening, as well as prostate cancer in males (94, 95). Consumption of As in food products above safe level causes cardiovascular diseases, developmental anomalies, hearing defects, carcinoma, and hematologic disorders (96, 97). Hg is known to cause permanent damage to the central nervous system in children (98, 99). Effects such as heart function alteration, leukemia, kidney damage, neurocognitive defects and neuromotor disabilities have been reported in children exposed to Hg in sea food (100–102). Besides, Hg could affect children during any stage of development including maternal exposure particularly from methyl mercury (MeHg) species (87). Exposure to Cr could affect the functions of the heart, hematological parameters, kidneys, liver, and the central nervous system (103). Therefore, it is suggested that regular monitoring of heavy metals accumulation levels in aquatic environments and fish be conducted to safeguard public health (104, 105).

**Table 1:** Recommended values of some heavy metal elements by the food and agriculture organization FAO (106).

**Heavy metal**	**Value (wet weight)**	**Value (dry weight)**
Cr	0.15–1.0 ppm	0.65–4.35 ppm
Zn	30.0 ppm	130.43 ppm
Mn	1.00 ppm	4.35 ppm
Fe	100.00 ppm	434.78 ppm
Co	0.04–0.26 ppm	0.17–1.13 ppm
Cu	30.00 ppm	130.43 ppm
Se	1.00 ppm	4.35 ppm
Hg	0.50 ppm	2.17 ppm
Pb	0.50 ppm	2.17 ppm
Ni	80.00 ppm	347.82 ppm
As	1.00 ppm	4.35 ppm

Fish, also being a perishable product, is vulnerable to fermentation and decomposition resulting in biogenic amines (BA) that threaten fish safety for consumption. BA refer to toxicants non-volatile amines resulting from amino acids decarboxylation (107). They are produced either by proteolytic activities of certain microorganisms or naturally during the metabolism of related precursor amino acids (108). However, in fish, histamine (HIS), cadaverine (CAD), and putrescine (PUT) are the only biogenic amines of concern when it comes to food safety and quality control (107). HIS is a monoamine produced from histidine precursor amino acid *via* a one-step decarboxylation reaction (108). CAD is a diamine produced from lysine and putrescine *via* a decarboxylation reaction (109). PUT is also a diamine but is produced either through a single-step decarboxylation from agmatine and ornithine, or indirectly after arginine hydrolysis (110). Although BA, at their physiological levels, play important roles in various cells process such as cell growth, gene expression, and tissue repair (111, 112), their ingestion at higher levels, although unlikely, could pose serious health hazards like symptoms of histamine poisoning including anaphylaxis, hypertension, nervous manifestation, and even death (113). Besides, Doeun et al. (114) reported that CAD and PUT could give way to gastric cancer during its conversion into carcinogenic N-nitroso compounds by microorganisms present in the digestive tract. Furthermore, in Europe, consumption of fish containing elevated levels of BAs was associated with widespread cases of intoxication (European Food Safety Authority EFSA (115) Therefore, it is very important that fish products are screened for BAs before administered for consumption to safeguard public health. This can be done 2-fold: maintaining high level of hygiene during fish harvesting, storage, processing and distribution to consumers, and by controlling total mesophilic (TMC) and total psychrophilic (TPsC) bacterial counts in fish products. El-Ghareeb et al. (109) observed a positive correlation between total BAs and TMC, suggesting that microorganisms play a major role in contaminating fish products with BAs.

### Contextualizing Research on Children and Food Marketing

To ensure the growth, health and development of children to their full potential, adequate nutrition during infancy and early childhood is essential. The most effective interventions to improve child health is through optimal infant and young child feeding practices (116). Poor diets will drive malnutrition in early childhood and millions of children are eating too little of what they need, and millions are eating too much of what they do not need which is the main risk factor for the global burden of diseases (117). According to Unicef (117) malnutrition, which is an umbrella term for both excess consumption of nutrients (overnutrition), inadequate consumption of nutrients (undernutrition) or micronutrient deficiency (“hidden hunger”), is primarily caused by a suboptimal diet. The burden of child undernutrition remains a global threat, with 21.3 percent of children under the age of 15 years stunted, 6.9 percent wasted and 340 million suffering from micronutrient deficiencies (3). It is reported by (118) and (119) that in addition to contributing to greater dietary diversity and boosting the micronutrient intake of women of reproductive age, the consumption of aquatic foods in the first 1,000 days of life from conception to a child's second birthday is associated with positive birth outcomes, a better nutrient composition of breastmilk, reduced stunting and a decline in the prevalence of severe acute malnutrition. It is also attested that eating fish early in life can promote positive behavioral and mental health outcomes and prevent certain allergies, such as asthma, eczema, and allergic rhinitis (41).

Aquatic foods, especially aquatic animals, have long been valued as a rich source of animal protein and thus, considered a key constituent of nutritious diets (120) but the policies on aquatic foods tend to focus primarily on production, economic efficiency, resource management, environment and climate issues whilst paying less attention to value chains and the contribution of aquatic foods to people's nutrition and health. Ahern et al. (121) recommended that aquatic foods are part of the solution to building resilient food systems and sustainable healthy diets for all, but for this to be fully achieved, they need to be available, accessible, affordable and desired. The nutritional value and health benefits of the fishes are unrecognized and undervalued. Despite a lot of benefits in the health of humans particularly the children, some people are still unaware of these benefits (122). The contribution of capture fisheries for instance to diet quality is poorly understood in most contexts, particularly where small-scale fisheries remain undocumented and overlooked in both fisheries and food system development (123, 124) hence limiting the nuanced assessment of fisheries contribution to diet quality of children under 12 years of age which is the critical age at which interventions have the greatest long-term effects for growth and health (117, 125). In contrast, Crona et al. (126) and HLPE (127) records that fish and other aquatic foods are gaining attention for their potential to efficiently provide two fundamental components of sustainable, nutritious food systems. Fish from inland fisheries are an important source of animal source foods (ASF) in monotonous diets for children in the sub-Saharan Africa and Asia (128), especially in land-locked African countries such as Malawi (129, 130) and Zambia (131). Therefore, there is a need to realize the importance of fish for human nutrition, in addition to its role in reducing poverty and hunger. This will ensure a greater impact by improving the nutritional status of children.

## Conclusion

The nutritional benefits of fish consumption in children has been reviewed. Our findings show that fish is an important animal protein source and its consumption is likely to increase over the coming years. This will be driven primarily by population increase and the demand for healthy and high-quality protein for human nutrition. The polyunsaturated fatty acids (PUFAs) that are highly present in fish play an important physiological role in the growth and development of fetuses, newborns, and children's brains. As a result, they should be provided in the diets of children for normal development. Besides, *in situations* including auto-immune illnesses diseases, PUFAs have been found to enhance blood flow, minimize chronic inflammation and decrease coronary artery disease.

## Author Contributions

SM and KN wrote the draft manuscript. MA-T reviewed the manuscript. HK designed the study and wrote and formulated the objectives, as well as reviewed the manuscript. All the authors read and approved the final manuscript.

## Funding

This research was done jointly by Wageningen University, which provided support in the form of a postdoctoral fellowship, and the WorldFish Center's CGIAR Research Program (Trust Fund) on Fish Agri-Food Systems (FISH) [F11194].

## Conflict of Interest

The authors declare that the research was conducted in the absence of any commercial or financial relationships that could be construed as a potential conflict of interest.

## Publisher's Note

All claims expressed in this article are solely those of the authors and do not necessarily represent those of their affiliated organizations, or those of the publisher, the editors and the reviewers. Any product that may be evaluated in this article, or claim that may be made by its manufacturer, is not guaranteed or endorsed by the publisher.
